# Mechanistic biomarkers provide early and sensitive detection of acetaminophen-induced acute liver injury at first presentation to hospital

**DOI:** 10.1002/hep.26294

**Published:** 2013-07-02

**Authors:** Daniel J Antoine, James W Dear, Philip Starkey Lewis, Vivien Platt, Judy Coyle, Moyra Masson, Ruben H Thanacoody, Alasdair J Gray, David J Webb, Jonathan G Moggs, D Nicholas Bateman, Christopher E Goldring, B Kevin Park

**Affiliations:** 1 MRC Centre for Drug Safety Science, Department of Molecular & Clinical Pharmacology, University of LiverpoolLiverpool, UK; 2 NPIS Edinburgh, Royal Infirmary of EdinburghUK; 3 University of Edinburgh/British Heart Foundation Centre for Cardiovascular ScienceEdinburgh, UK; 4 Emergency Medicine Research Group, Department of Emergency Medicine, Royal Infirmary of EdinburghEdinburgh, UK; 5 NPIS Newcastle, Regional Drugs and Therapeutics Centre, School of Clinical and Laboratory Sciences, University of NewcastleNewcastle upon Tyne, UK; 6 Discovery & Investigative Safety, Preclinical Safety, Novartis Institutes for Biomedical Research (NIBR)Basel, Switzerland

## Abstract

**A**cetaminophen overdose is a common reason for hospital admission and the most frequent cause of hepatotoxicity in the Western world. Early identification would facilitate patient-individualized treatment strategies. We investigated the potential of a panel of novel biomarkers (with enhanced liver expression or linked to the mechanisms of toxicity) to identify patients with acetaminophen-induced acute liver injury (ALI) at first presentation to the hospital when currently used markers are within the normal range. In the first hospital presentation plasma sample from patients (n = 129), we measured microRNA-122 (miR-122; high liver specificity), high mobility group box-1 (HMGB1; marker of necrosis), full-length and caspase-cleaved keratin-18 (K18; markers of necrosis and apoptosis), and glutamate dehydrogenase (GLDH; marker of mitochondrial dysfunction). Receiver operator characteristic curve analysis and positive/negative predictive values were used to compare sensitivity to report liver injury versus alanine transaminase (ALT) and International Normalized Ratio (INR). In all patients, biomarkers at first presentation significantly correlated with peak ALT or INR. In patients presenting with normal ALT or INR, miR-122, HMGB1, and necrosis K18 identified the development of liver injury (n = 15) or not (n = 84) with a high degree of accuracy and significantly outperformed ALT, INR, and plasma acetaminophen concentration for the prediction of subsequent ALI (n = 11) compared with no ALI (n = 52) in patients presenting within 8 hours of overdose. *Conclusion*: Elevations in plasma miR-122, HMGB1, and necrosis K18 identified subsequent ALI development in patients on admission to the hospital, soon after acetaminophen overdose, and in patients with ALTs in the normal range. The application of such a biomarker panel could improve the speed of clinical decision-making, both in the treatment of ALI and the design/execution of patient-individualized treatment strategies.

Acetaminophen (paracetamol) overdose is a common reason for hospital admission and accounted for 38,000 emergency hospital admissions in the financial year 2010-2011 in England alone.^1^ Antidote treatment with acetylcysteine (AC) takes at least 20 hours to complete, which results in significant hospital bed occupancy (around 47,000 bed days per year in England). Also, adverse reactions to AC are common; up to 45% of treated patients develop adverse effects, which includes mainly vomiting and nausea.^2^ However, in a study of 362 patients, 15% experienced anaphylactoid reactions that required cessation of AC^3^ and there have been incidences of fatalities.^4^ Therefore, careful patient selection is required to maximize the balance of risk to benefit. The decision to start treatment is predominately based on the dose of acetaminophen ingested and a timed blood acetaminophen concentration, because the majority of patients present to the hospital soon after drug ingestion, before acute liver injury (ALI) can be diagnosed, or confidently excluded, using current blood-based biomarkers such as alanine aminotransaminase (ALT). This early clinical uncertainty regarding the presence of liver cell injury prevents treatment being individualized, potentially leading to patients being overtreated with a time-consuming and potentially harmful antidote or undertreated, with increased risk of ALI.

New biomarkers or panels of biomarkers that report acetaminophen-induced ALI at the earliest possible timepoint are required. Moreover, their potential to provide insight into the underlying mechanistic basis of ALI is increasingly recognized as fundamental to efforts in translational research and patient treatment stratification.^5^ Such biomarkers could permit therapy to be targeted to patients at high risk of adverse outcome or allow the early and safe discharge of well/low-risk groups.

Recent preclinical reports, from our group and elsewhere, of acetaminophen-induced ALI have identified microRNA-122 (miR-122), high mobility group box-1 (HMGB1), keratin-18 (K18, both caspase-cleaved and full-length), and glutamate dehydrogenase (GLDH) as sensitive markers of hepatotoxicity.^6^ These blood-based biomarkers have been applied to the clinical setting of severe acetaminophen hepatotoxicity in patient cohorts from both the UK and USA and some have been reported to more accurately predict patient prognosis and outcome than currently used clinical chemistry parameters; in particular, the acetylated isoform of HMGB1 has been shown to provide superior prognostic potential than ALT activity.^8^

In addition to the early diagnosis of acetaminophen-induced liver injury in rodent models, and the prediction of patient prognosis once hepatotoxicity is established, these biomarkers can provide enhanced mechanistic information regarding the underlying basis of acetaminophen poisoning. HMGB1 is reflective of cell necrosis and activated immune cells.^11^ The caspase-cleaved form of K18 has been shown to reflect cell apoptosis and the full-length variant, necrosis.^12^*^13^ GLDH is highly expressed in the mitochondria of the liver and can report mitochondrial dysfunction, while miR-122 has been shown to be an earlier marker of acetaminophen-induced liver injury than ALT in mice and provides enhanced hepatic specificity over current clinical biomarkers.^7^,^8^ However, despite their clinical potential, few data exist in man on the value of these markers in reporting acetaminophen-induced liver injury earlier than currently used approaches.

Therefore, we assessed the potential utility of miR-122, HMGB1, K18, and GLDH to serve as early biomarkers of acetaminophen-induced ALI. We hypothesized that based on superior tissue specificity, earlier time of release, a mechanistic association or increased sensitivity with respect to bioanalysis, elevations in these blood-based biomarkers could identify patients with ALI at first presentation to the hospital emergency department.

## Patients and Methods

### Patients

Patients (total N = 129) were recruited from the Royal Infirmary of Edinburgh, UK (N = 107) and the Royal Victoria Infirmary, Newcastle-Upon-Tyne, UK (N = 22). Full informed consent was obtained; the study was approved by the local research Ethics Committee. Inclusion criteria were: adult with a clear history of single excess acetaminophen ingestion and a timed blood acetaminophen concentration that was judged by the treating physician to necessitate hospital admission for intravenous AC therapy, as per UK guidelines at the time of study (www.toxbase.org). Exclusion criteria were: patients detained under the Mental Health Act; patients with known permanent cognitive impairment; patients with a life-threatening illness; unreliable history of acetaminophen overdose; patients who take anticoagulants therapeutically or have taken an overdose of anticoagulants; and patients who, in the opinion of the responsible clinician/nurse, were unlikely to complete the full course of AC. All patients completed the full course of AC treatment. In all, 97 patients were participants in the Scottish and Newcastle Antiemetic Pretreatment for Acetaminophen Poisoning study (SNAP, EudraCT number 2009-017800-10), a randomized clinical trial designed to assess if ondansetron is effective in reducing nausea and vomiting in patients poisoned with acetaminophen treated with AC. As a predefined part of the SNAP trial protocol, blood samples were collected for biomarker evaluation. In all 129 study participants, blood samples were collected at the time of first presentation to the hospital, before AC (and for SNAP study participants, ondansetron or placebo) therapy had commenced. Plasma was separated and the samples were stored at −80°C until analysis. For all study participants, demographic data and blood results were recorded. ALI was defined as peak serum ALT activity greater than 3× the upper limit of the normal range (ULN = 50 IU/L), the UK indication for continuing AC therapy after completion of the initial regimen (www.toxbase.org) at the time of the study. Plasma biomarkers were determined as previously described for miR-122,^10^ HMGB1,^9^ K18,^9^ and GLDH.^15^ For miR-122 measurement (delta Ct), let-7d provided biological standardization. The persons measuring the biomarkers were blinded to the biochemistry results of the patients.

### Statistical Analysis

Data are presented as median and range or interquartile range (IQR). Each dataset was analyzed for nonnormality using a Shapiro-Wilk test. For two nonnormal datasets, comparisons were made using the Mann-Whitney *U* test. The Kruskall-Wallis test was used to determine significance between more than two nonnormal sample groups. All calculations were performed using StatsDirect statistical software. For correlative analysis, Pearson's correlation test, R^2^, and receiver operator characteristic (ROC) curve analysis were carried out using GraphPad PRISM software. Results were considered significant when *P* < 0.05. The funding sources had no influence over the study design, data analysis, or article production.

## Results

### Biomarker Concentration at Hospital Presentation Correlated With Subsequent Liver Injury

In our cohort, there were 51 men (40%) and 78 women (60%) with a median age of 34 years (Table1). Fifty-four percent of this cohort had at least one risk factor for acetaminophen-induced liver injury (at the time of study these risk factors were considered to be: malnourishment, nutritional deficiency, or being at risk of hepatic enzyme induction, such as due to chronic alcohol ingestion). Seventy-three percent of the total cohort had taken one extra medication with the acetaminophen overdose; the most common coingested drug class was nonopioid analgesics. The median time (IQR) from acetaminophen ingestion to first blood sample collection was 8 hours (6-15 hours). The median admission blood acetaminophen concentration was 120 mg/L (IQR, 63-179). From timed plasma acetaminophen concentrations, the Rumack-Matthew nomogram indicated that all 129 patients required AC therapy. The median (IQR) clinical chemistry values at first hospital presentation were: serum creatinine: 67 μmol/L (60-82), bilirubin: 5 μmol/L (7-11), serum ALT activity: 22 IU/L (16-58), serum ALP activity: 75 IU/L (61-95), serum GGT activity: 24 IU/L (15-46) and an International Normalized Ratio (INR) of 1.0 (0.9-1.1). The number of patients presenting with a serum ALT activity less than 3× ULN was 98 (76%), with 31 (24%) patients more than 3× ULN. The number of patients presenting with an INR ratio <1.5 was 107 (85%) with 19 (15%) presenting with an INR >1.5. Three patients did not have an INR determined from the presentation sample. No patients required liver transplantation or developed encephalopathy.

**Table 1:** Clinical Parameters of the Acetaminophen Overdose Patient Cohort

Number	129
Sex (male:female)	51:78
Age (years)	34 (24.5-47.0)
Risk Factors (% of cohort)	54
Amount of acetaminophen ingested (g)	18.0 (13.0-25.0)
Time from ingestion to first blood sample (hr)	8.0 (6.0-15.0)
Admission acetaminophen concentration (mg/L)	120.0 (63.0-179.0)
Time from ingestion to start of AC treatment (hr)	10.0 (7.0-19.0)
Admission serum creatinine (μmol/L)	67.0 (60.0-81.5)
Admission bilirubin (μmol/L)	5.0 (7.0-11.0)
Admission ALT activity (IU/L)	22.0 (16.0-57.5)
Admission ALP activity (IU/L)	75.0 (60.5-94.5)
Admission GGT activity (IU/L)	24.0 (15.0-46.0)
Admission INR	1.0 (0.9-1.1)
Number with admission ALT < ULN	98
Number with peak ALT > 3x ULN	31
Number with peak ALT > 1,000 U/L	20
Number with admission INR < 1.5	107
Number with peak INR > 1.5	19

Various clinical parameters of the patient cohort who present early after acetaminophen ingestion. AC, acetyl-cysteine; ALT, alanine aminotransferase; ALP, alkaline phosphatase; GGT, gamma glutamyl transpeptidase; INR, International Normalized Ratio; ULN, upper limit of normal.

**Figure 1:**
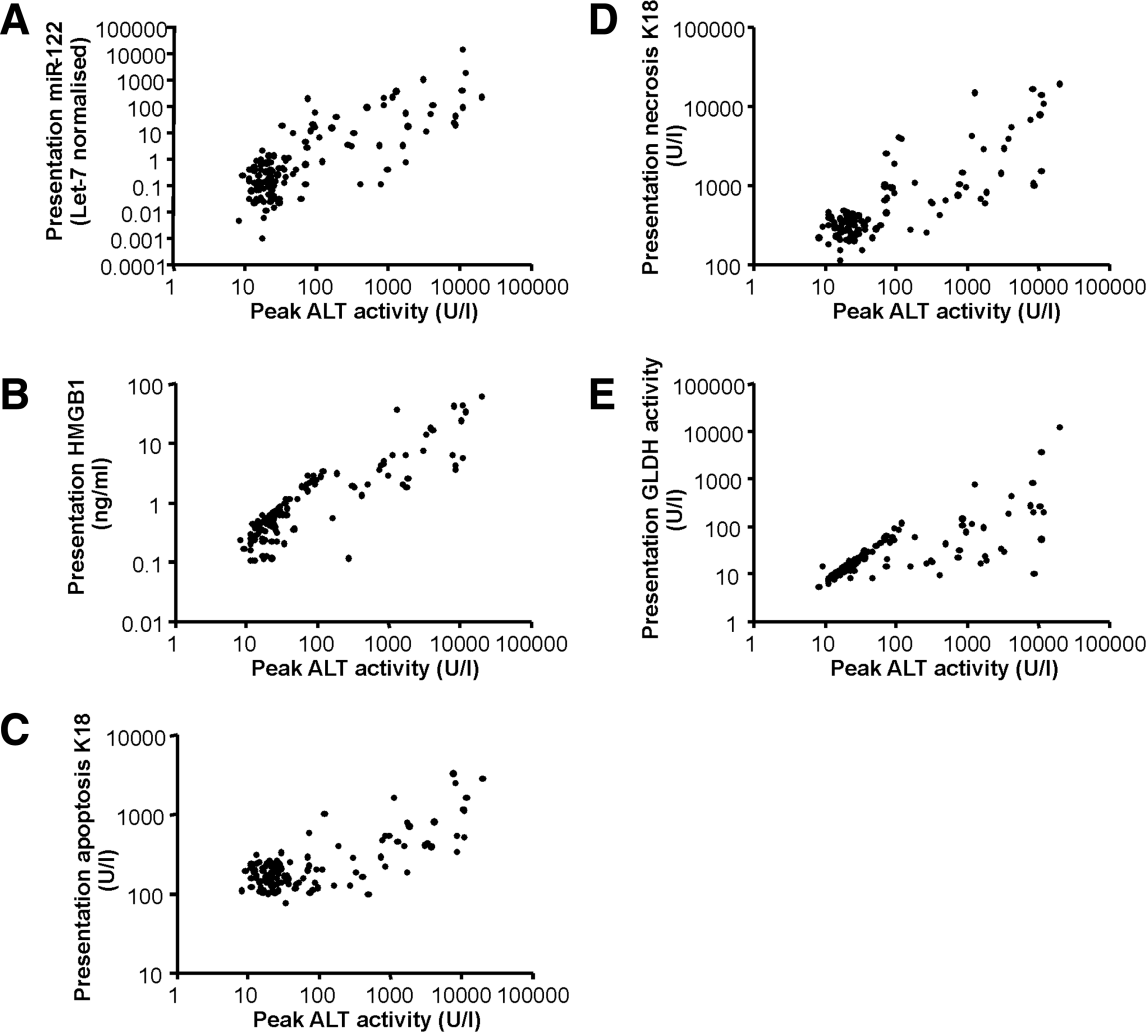
Plasma biomarker values at presentation to the hospital emergency department correlate with peak ALT activity. (A) miR-122, (B) total HMGB1, (C) apoptosis K18, (D) necrosis K18, and (E) GLDH activity were correlated against peak ALT activity in patients who presented early after acetaminophen overdose (<24 hours, n = 129).

We measured miR-122, HMGB1, apoptosis K18, necrosis K18, and GLDH activity in plasma obtained at the point of hospital admission, before AC treatment had begun, but when a timed blood acetaminophen concentration had indicated the requirement for AC therapy. We performed a correlation analysis on values obtained from each individual marker against the peak serum ALT activity during patient hospitalization. The presentation serum miR-122, HMGB1, apoptosis K18, necrosis K18, and GLDH activity values all significantly correlated with peak ALT activity values (*P* < 0.0001, Fig. 1A-E). The correlation coefficients (R^2^) were 0.14, 0.67, 0.57, 0.59, and 0.45 and the Pearson R values (95% confidence interval [CI]) were 0.37 (0.21-0.52), 0.82 (0.75-0.87), 0.75 (0.67, 0.82), 0.77 (0.69-0.83), and 0.67 (0.56-0.76) for miR-122, HMGB1, apoptosis K18, necrosis K18, and GLDH activity, respectively.

**Figure 2:**
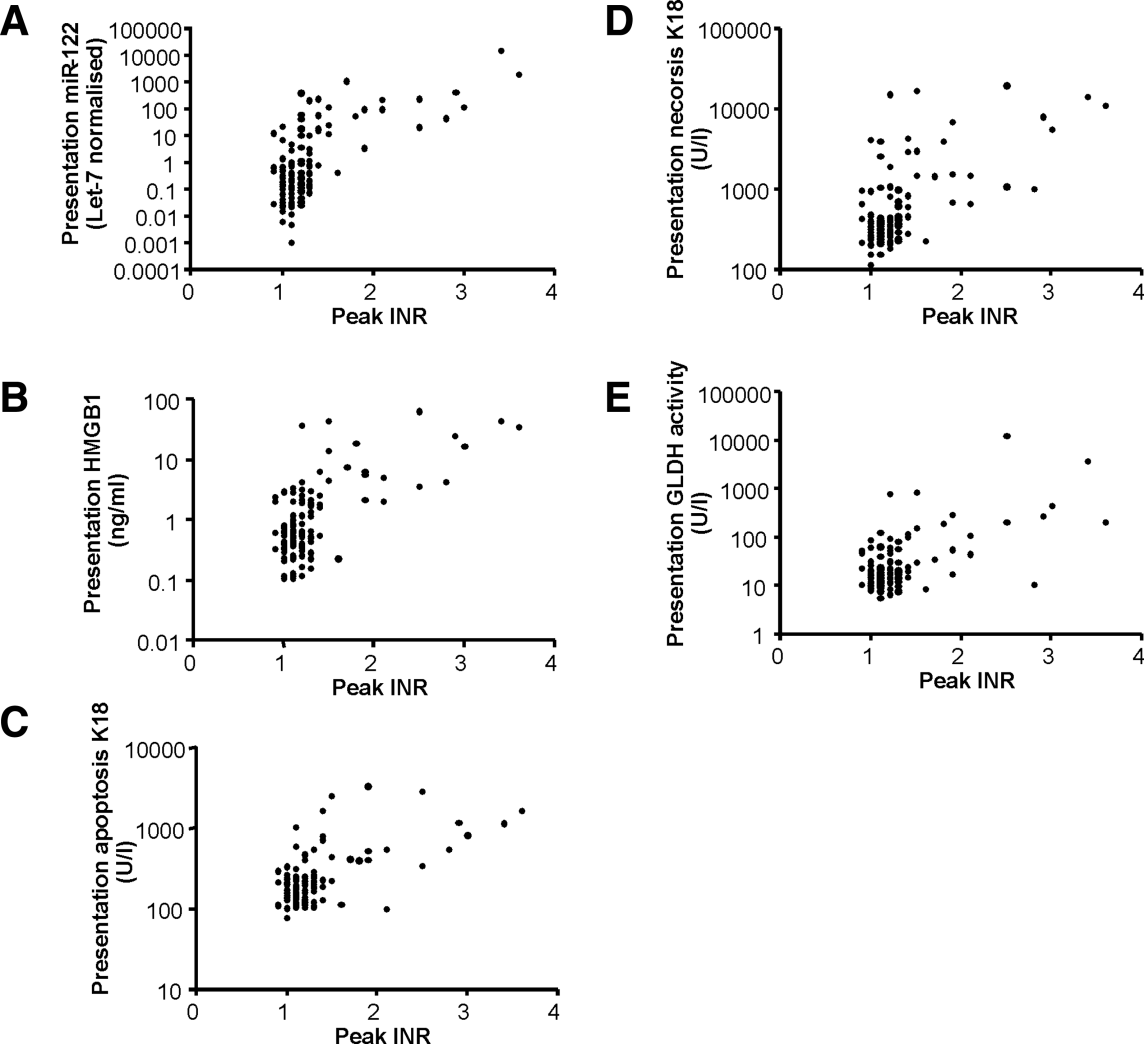
Plasma biomarker values at presentation to the hospital emergency department correlate with peak INR. (A) miR-122, (B) total HMGB1, (C) apoptosis K18, (D) necrosis K18, and (E) GLDH activity were correlated against peak INR in patients who presented early after acetaminophen overdose (<24 hours, n = 129).

**Figure 3:**
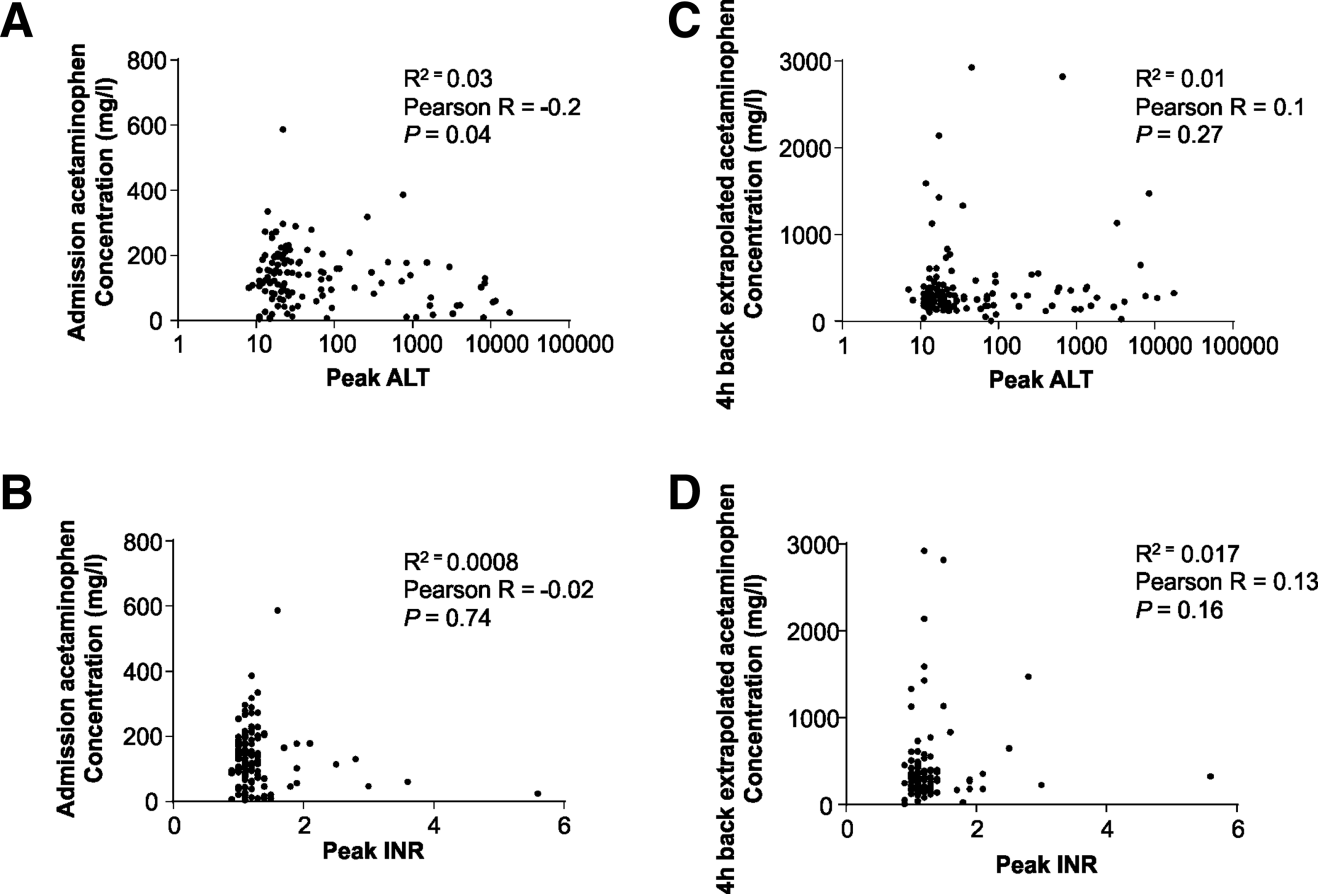
Correlation of admission and 4-hour back-extrapolated plasma acetaminophen concentration with peak ALT and INR. Plasma (A) peak ALT activity and (B) peak INR were correlated with admission acetaminophen concentration for all patients (n = 129). Plasma (C) peak ALT activity and (D) peak INR were correlated with 4-hour back-extrapolated acetaminophen concentration for all patients (n = 129). Correlation coefficient, Pearson R values, and statistical significance are indicated.

We performed a correlation analysis from individual marker values at first presentation against the peak INR value during patient hospitalization. The presentation values for plasma miR-122, HMGB1, apoptosis K18, necrosis K18, and GLDH activity all significantly correlated with peak INR values (*P* < 0.0001, Fig. 2A-E). The correlation coefficients (R^2^) were 0.24, 0.42, 0.29, 0.34, and 0.13 and the Pearson R values (95% CI) were 0.49 (0.34-0.61), 0.64 (0.53-0.74), 0.54 (0.40, 0.65), 0.59 (0.46-0.69), and 0.37 (0.21-0.51) for miR-122, HMGB1, apoptosis K18, necrosis K18, and GLDH activity, respectively. Presentation and 4-hour back-extrapolated plasma acetaminophen concentration did not significantly correlate with either peak ALT activity or peak INR (Fig. 3).

### In Patients With a Normal ALT Activity on First Hospital Presentation, Serum miR-122, HMGB1, Apoptosis K18, Necrosis K18, and GLDH Activity Are Higher in Those Who Develop ALI

**Figure 4:**
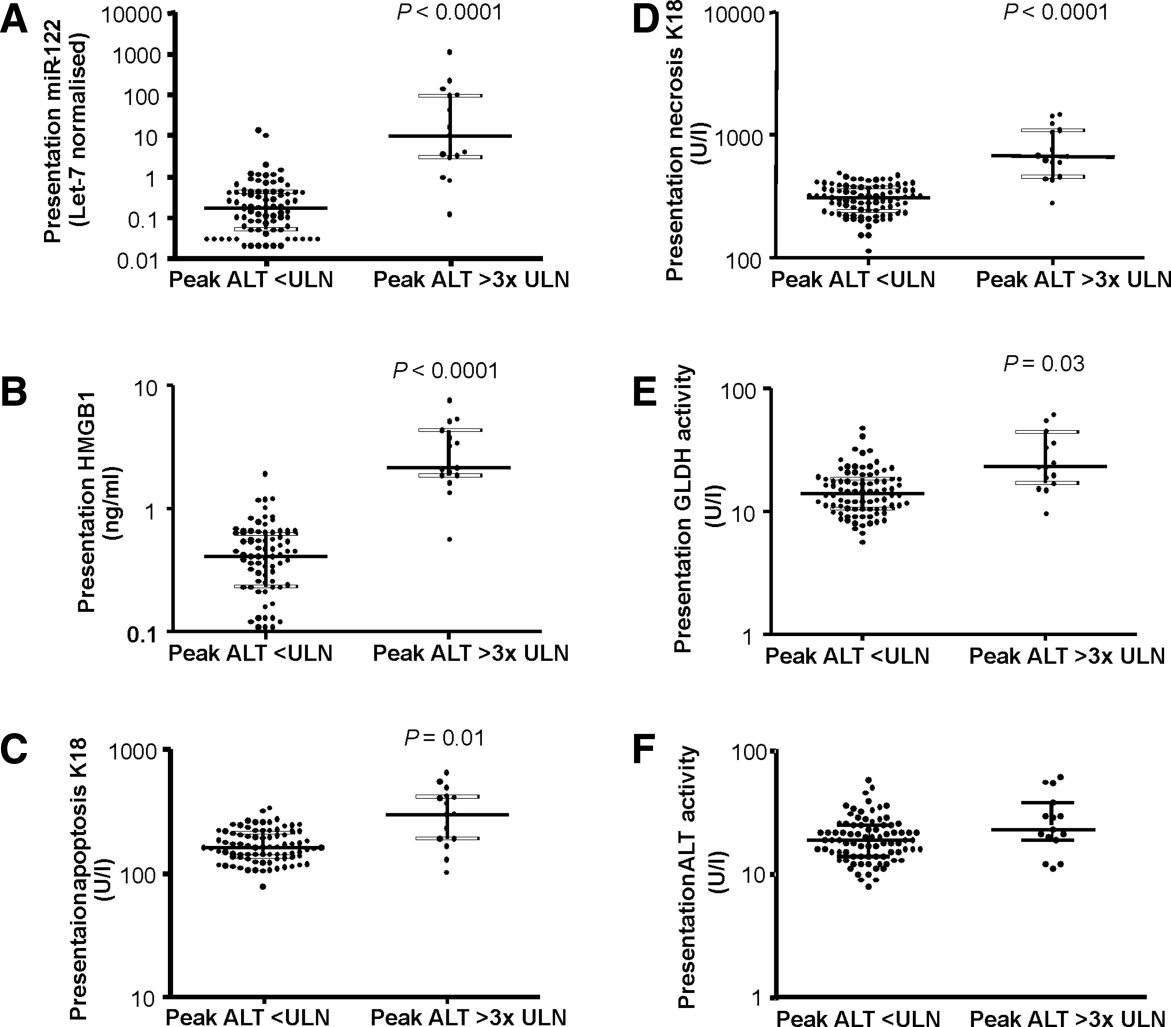
Plasma biomarker values are elevated in patients with normal liver function tests who subsequently develop ALI. (A) miR-122, (B) total HMGB1, (C) apoptosis K18, (D) necrosis K18, (E) GLDH activity, and (F) ALT activity were quantified in plasma from patients who presented within 24 hours of a acetaminophen overdose and had an ALT activity level within the normal range (<50 IU/L) at presentation (n = 98). Presentation values for miR-122, total HMGB1, apoptosis K18, necrosis K18, GLDH, and ALT are compared between patients who do not (n = 83) and do (n = 15) develop ALI, as defined by an ALT activity rising above 3× ULN. Data are given as median (IQR) and statistical significance is recorded on each figure as required for each biomarker.

From our cohort of 129 acetaminophen overdose patients, we focused on 98 with an ALT activity <3× ULN with a median (IQR) value of 21 IU/L (15-29) at first presentation to determine if the new biomarkers are more sensitive than ALT. Of these patients, 15 (15%) developed liver injury (>3× ULN ALT activity; median peak ALT [IQR] 445 IU/L [224-1187]) during their hospitalization, whereas 83 (85%) patients did not develop liver injury at any time during the study (<3× ULN ALT activity; median peak ALT [IQR] 19.0 IU/L [14-25]). First presentation concentration for each biomarker was significantly higher in patients who developed ALI than in those who did not (Fig. 4A-E). The median values (IQR) between the group of patients who later developed injury and the group that did not develop toxicity were 9.8 (2.8-96.0) versus 0.17 (0.05-0.43) for miR-122 (*P* < 0.0001), 2.14 (1.9-4.3) versus 0.41 (0.23-0.63) ng/mL for HMGB1 (*P* < 0.0001), 298.4 (189.8-648.1) versus 161.1 (133.2-337.4) U/L for apoptosis K18 (*P* = 0.01), 672.0 (460.1-1098.0) versus 312.1 (237.2-382.0) U/L for necrosis K18 (*P* < 0.0001), and 23.00 (16.8-44.8) versus 13.9 (10.4-18.4) for GLDH activity (*P* = 0.03).

**Figure 5:**
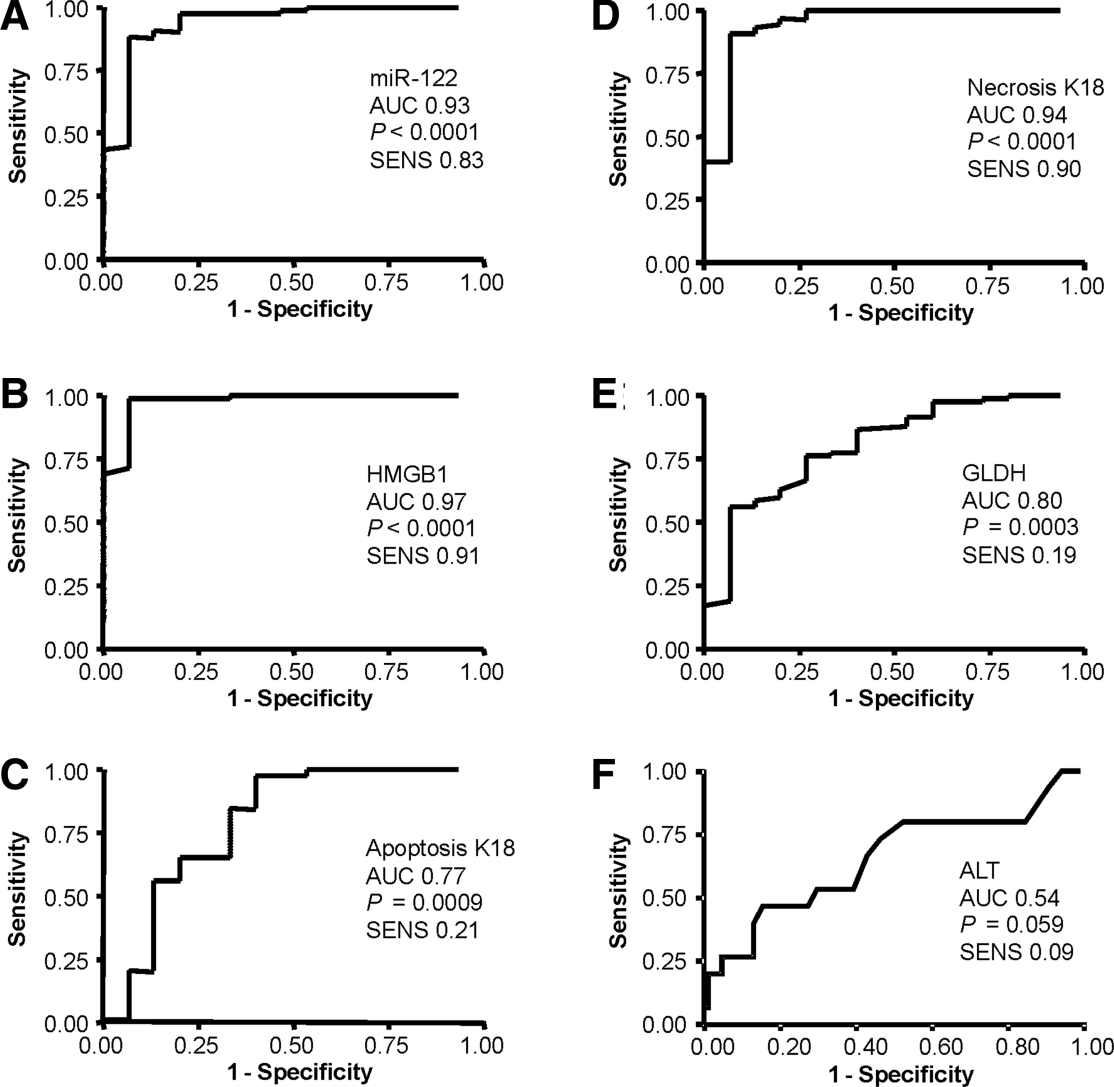
ROC curve analysis supports the potential for novel biomarkers to predict the development of ALI. ROC analysis was calculated to determine the potential for plasma (A) miR-122, (B) total HMGB1, (C) apoptosis K18, (D) necrosis K18, (E) GLDH activity, and (F) ALT activity to predict the development of ALI following presentation <24 hours following acetaminophen overdose. All patients (n = 98) presented with ALT activity within the normal range (<50 IU/L) and then either subsequently stayed within the normal range (n = 83) or developed ALI as defined by an ALT activity rising above 3× ULN (n = 15). AUC, statistical significance, and sensitivity (SENS) at 90% specificity is recorded on each figure as required.

We performed ROC curve analysis on these groups to provide a robust test of sensitivity and specificity of our set of markers (Fig. 5A-E). Three of these markers, miR-122, HMGB1, and necrosis K18 performed with high AUC values (sensitivity at 90% specificity, 95% CI, *P* < 0.0001); 0.93 (0.83, 0.86-1.0, *P* < 0.0001), 0.97 (0.91, 0.91-1.0, *P* < 0.0001), and 0.94 (0.90, 0.87-1.0, *P* < 0.0001), respectively, suggesting that these markers could provide the greatest separation between patients with and without ALI at a time when serum ALT activity was normal. The AUC values (and sensitivity at 90% specificity, 95% CI) for apoptosis K18 and GLDH were 0.77 (0.21, 0.63-0.95, *P* = 0.0009) and 0.80 (0.19, 0.68-0.93, *P* = 0.0003), respectively. The positive predictive values (negative predictive values) were 70.6% (97.5%), 85.7% (97.6%), 70.1% (92.1%), 73.3% (96.3%), and 50.0% (88.9%) for serum miR-122, HMGB1, apoptosis K18, necrosis K18, and GLDH, respectively (Fig. 5). Presentation ALT activity itself was a poor predictor of the development of acute liver injury, with an AUC of 0.54 (95% CI 0.29-0.71) and a sensitivity of 0.09 at 90% specificity (Fig. 5F).

### Circulating miR-122, HMGB1, Apoptosis K18, Necrosis K18, and GLDH Activity Are Superior to Serum ALT Activity in Identifying ALI in Patients Whose First Blood Sample Was Within 8 Hours of Overdose

From our cohort of 129 acetaminophen overdose patients, we next focused on 63 with a first blood sample taken within 8 hours of drug ingestion to determine whether the new biomarkers detect early ALI. Of these patients, 11 (17%) developed liver injury (>3× ULN serum ALT activity; median peak ALT [IQR] 487.0 IU/L [266.0-942.0]) during their hospitalization, whereas 52 (83%) patients did not develop liver injury at any time during the study (serum ALT activity remained <3× ULN). First presentation concentration for each biomarker was significantly higher in patients who developed ALI than those who did not. The median values (IQR) for patients who developed ALI and for patients who did not were 3.69 (0.43-96.0) versus 0.21 (0.07-0.81) for miR-122 (*P* = 0.002), 2.14 (1.36-4.33) versus 0.54 (0.34-0.86) ng/ml for HMGB1 (*P* < 0.001), 402.7 (129.0-487.0) versus 159 (126.0-206.0) U/L for apoptosis K18 (*P* = 0.02), 685.0 (435.0-1098.0) versus 328 (242.0-428.0) U/L for necrosis K18 (*P* = 0.002), and 33.0 (16.8-61.0) versus 16.3 (12.0-22.0) for GLDH activity (*P* = 0.03). In contrast, there was no difference in serum ALT activity; the median values (IQR) for patients who developed injury and for patients that did not develop ALI were: 21 (12.0-56.0) U/L versus 21 (16.5-31.5) U/L. We performed an ROC curve analysis (Table2) which demonstrated high predictive performance of each of the biomarkers to reflect the subsequent development of liver injury or not. Consistent with there being no difference in serum ALT activity between groups, the ROC curve analysis supported this finding (Table2). In addition, the median (IQR) plasma acetaminophen concentration was not significant between patients who did and did not develop ALI; 177.0 (139.0-208.0) versus 165 (123.0-204.8) mg/L (*P* = 0.691). Furthermore, back extrapolation of the presentation acetaminophen concentration to 4 hours was also not significant between patients who did and did not develop ALI; 179.0 (165.0-354.0) versus 269.5 (179.5-351.0) mg/L (*P* = 0.449). Consistent with there being no difference in plasma acetaminophen concentration between groups, the ROC curve analysis also supported this finding (Table2). Table3 also summarizes the predictive performance for miR-122, HMGB1, K18, and GLDH quantified from the first presentation sample to reflect ALI in patients admitted to the hospital emergency room later than 8 hours following overdose (n = 62). Consistent with the observations from the <8-hour cohort, these biomarkers display a strong diagnostic capability compared to ALT activity and plasma acetaminophen concentration for patients who develop ALI defined as a peak ALT activity >3× ULN (n = 20) and those who do not (n = 42) (Table3).

**Table 2:** Receiver Operator Characteristic (ROC) Curve Analysis Supports the Potential for Novel Biomarkers to Predict the Development of ALI Within 8 Hours of Acetaminophen Overdose

Biomarker	ROC-AUC (95% CI)	SENS (95% C.I)	PPV	NPV	*P* Value
miR-122	0.80 (0.61-1.0)	0.45 (0.29-0.60)	72.7	86.5	0.002
HMGB1	0.83 (0.69-1.0)	0.63 (0.48-0.77)	90.7	86.5	< 0.001
Apoptosis K18	0.73 (0.51-0.96)	0.63 (0.48-0.76)	72.7	96.1	0.02
Necrosis K18	0.80 (0.68-0.97)	0.45 (0.20-0.76)	63.6	86.5	0.002
GLDH	0.71 (0.54-0.89)	0.31 (0.17-0.45)	45.4	88.4	0.03
ALT	0.52 (0.31-0.74)	0.09 (0.02-0.21)	36.3	84.6	0.80
[Acetaminophen] presentation	0.54 (0.38-0.72)	0.18 (0.09-0.30)	27.3	69.2	0.68
[Acetaminophen] 4hr extrapolated	0.57 (0.36-0.78)	0.18 (0.08-0.32)	45.5	28.8	0.44

ROC analysis was calculated to determine the potential for plasma miR-122, total HMGB1, apoptosis K18, necrosis K18, GLDH activity, ALT activity, admission acetaminophen concentration (above or below 200 mg/L) and 4-hour back-extrapolated acetaminophen concentration (above or below 200 mg/L) to predict the development of ALI following presentation <8 hours following acetaminophen overdose. All patients (n = 63) presented with ALT activity within the normal range (<50 IU/L) and then either subsequently stayed within the normal range (n = 52) or developed ALI as defined by an ALT activity rising above 3x ULN (n = 11). ROC-AUC (area under the curve with 95% CI), sensitivity (SENS with 95% CI) at 90% specificity, positive (PPV) and negative (NPV) predictive value and statistical significance are given.

**Table 3:** Receiver Operator Characteristic (ROC) Curve Analysis Supports the Potential for Novel Biomarkers to Predict the Development of ALI in Patients Presenting Later Than 8 Hours After an Acetaminophen Overdose

Biomarker	ROC-AUC (95% CI)	SENS	PPV	NPV	*P* Value
miR-122	0.98 (0.95-1.0)	0.95 (0.83-0.99)	100.0	95.2	< 0.0001
HMGB1	0.99 (0.96-1.0)	0.91 (0.77-0.99)	100.0	90.4	< 0.0001
Apoptosis K18	0.91 (0.84-0.99)	0.65 (0.48-0.78)	80.0	90.4	< 0.0001
Necrosis K18	0.95 (0.90-1.0)	0.92 (0.81-0.98)	95.0	90.4	< 0.0001
GLDH	0.84 (0.71-0.97)	0.69 (0.52-0.82)	85.0	90.4	< 0.0001
ALT	0.96 (0.92-1.0)	0.76 (0.61-0.88)	80.0	90.4	< 0.0001
[Acetaminophen] presentation	0.60 (0.46-0.76)	0.28 (0.15-0.44)	0.0	92.8	0.1968
[Acetaminophen] 4hr extrapolated	0.54 (0.37-0.71)	0.11 (0.03-0.25)	85.0	30.9	0.6279

ROC analysis was calculated to determine the potential for plasma miR-122, total HMGB1, apoptosis K18, necrosis K18, GLDH activity, ALT activity, admission acetaminophen concentration (above or below 200 mg/L) and 4-hour back-extrapolated acetaminophen concentration (above or below 200 mg/L) to predict the development of ALI following presentation >8 hours following acetaminophen overdose. All patients (n = 62) either developed liver injury (peak ALT activity >150 U/L, n = 20) or did not (n = 42). ROC-AUC (area under the curve with 95% CI), sensitivity (SENS with 95% CI) at 90% specificity, positive (PPV) and negative (NPV) predictive value and statistical significance are given.

## Discussion

There is an urgent need to identify and validate biomarkers that show both improved sensitivity and hepatic specificity to assist the clinical management of acetaminophen poisoning. Current biomarkers to assess liver integrity include circulating markers of hepatocellular death and hepatic function assessed in combination.^5^ However, it is well documented that delayed presentation time, staggered overdose, and a lack of sensitivity of current clinical chemistry parameters remains a critical impediment to the treatment of acetaminophen overdose.^16^ Furthermore, there is an important and unmet need for the development of improved biomarkers that exhibit higher sensitivity and specificity for other forms of drug-induced liver injury (DILI). By building on our previous clinical investigations,^7^*^8^ and the preclinical reports from our laboratory and from others revealing that these biomarkers are more sensitive (with respect to time) indicators of changes in hepatic histology than ALT activity,^6^*^7^ we report for the first time that miR-122, HMGB1, and necrosis K18 represent early and sensitive biomarkers that predict the development of clinical acetaminophen hepatotoxicity.

In acetaminophen overdose patients with established liver failure, we have shown previously that the circulating level of HMGB1 and necrosis K18 predict which patients reach the Kings College Criteria (KCC) and which patients die / require a liver transplant, in particular the acetylated isoform of HMGB1.^9^*^10^ Moreover, our group have shown previously that miR-122 is 96% higher, on average, in patients meeting KCC versus patients who did not meet KCC; however, this did not meet statistical significance (*P* = 0.15).^10^ Within our previously published clinical investigations we sought to address the question as to whether or not these biomarkers could predict the outcome in patients with established acute liver injury; in the present study we now present the first data indicating that these biomarkers hold clinical utility to predict liver injury on presentation at the hospital emergency department.

Our conclusions are largely based on the observation and ROC statistical analysis that in this cohort of acetaminophen overdose patients, when liver function tests are within the normal range, each of miR-122, HMGB1, and necrosis K18 strongly predicted which patients subsequently developed liver injury and those who did not. Moreover, each biomarker significantly outperformed plasma acetaminophen concentrations measured in samples taken at the time of admittance, and 4-hour back-extrapolated acetaminophen determinations, for the prediction of the subsequent development of ALI. All the patients within our cohort received AC after the first blood sample was taken and no significant kidney injury was detected. The serum levels of each of the biomarkers investigated were not significantly elevated in patients who did not develop liver injury compared to healthy volunteers from our previous study.^9^*^10^ Immunohistochemistry data obtained from a mouse model of acetaminophen-induced ALI indicates that miR-122 is hepatocyte-specific and is lost progressively from necrotic areas of the liver during acetaminophen-induced liver injury (data not shown). The fact that miR-122 is greatly enriched in hepatocytes^14^ provides a significant advance in the development of novel biomarkers to identify and stratify acetaminophen hepatotoxicity early.

During the comparison of the presentation biomarker values between cohorts that did and did not subsequently develop liver injury, the potential of both GLDH and apoptosis K18 to reflect the development of liver injury was observed with less significance than miR-122, HMGB1, and necrosis K18. The induction of apoptosis by acetaminophen has been shown in animal models and also clinically during endstage acetaminophen hepatotoxicity by histology and the enzyme-linked immunosorbent assay (ELISA) detection of caspase-cleaved K18.^19^*^20^ However, necrotic cell death predominates and is a major factor in the development of acetaminophen-induced acute liver failure. The occurrence of apoptosis during acetaminophen hepatotoxicity is controversial. Studies reporting the identification of acetaminophen-induced apoptosis confirm that apoptosis is a minor event both clinically and *in vivo* and pan-caspase inhibitors do not protect against acetaminophen-induced liver injury.^6^*^9^ Rapid mitochondrial dysfunction is closely linked to hepatocyte necrosis and has been postulated as a reason for the lack of apoptosis during acetaminophen hepatotoxicity.^21^ Furthermore, using GLDH as a biomarker of mitochondrial dysfunction, recent reports have shown that GLDH activity closely associates with ALT activity during clinical acetaminophen hepatotoxicity,^8^ a finding replicated in our cohort. Therefore, the variable and minimal involvement of apoptosis during clinical and preclinical acetaminophen hepatotoxicity and the fact that GLDH activity is closely associated with ALT activity could, in part, explain the reduced sensitivity of these two biomarkers, compared to the others investigated here, to predict the development of ALI in a clinical setting.

Although the data presented here provide evidence that miR-122, HMGB1, and necrosis K18 represent sensitive and early biomarkers of clinical acetaminophen hepatotoxicity, without a tissue biopsy reflecting the actual degree of organ damage the observed quantification of these biomarkers could, in theory, overstate the degree of cell death. However, our data are consistent with those obtained in preclinical models, where quantitative circulating levels of a particular biomarker can be directly related to changes (or a lack of a change) in hepatic histology over time,^6^*^7^ and the finding that these biomarkers can report on the mechanistic basis of other DILIs in patient cohorts.^15^ Qualification is a common challenge for potential new biomarkers in man and it is important to note that ALT itself has never been fully qualified against human histology for drug-induced hepatotoxicity. Furthermore, the potential importance of the findings presented here is underpinned by the relatively heterogeneous nature of acetaminophen overdose cohort samples (particularly with respect to the amount of acetaminophen ingested and the time of presentation). We therefore believe that our data add to the growing body of evidence to support the ongoing clinical qualification of these markers of liver injury.

Within this continually evolving field, despite our evidence highlighting the sensitivity of these biomarkers in early overdose patients (<24 hours), little is still known regarding their kinetics in blood compared with ALT activity and their prognostic utility at later timepoints. However, our previously published clinical studies in the setting of acetaminophen overdose and heparin-induced liver injury reveal that these biomarkers appear to have a shorter half-life than ALT activity (24 hours compared to 48 hours) and return to baseline levels more rapidly than ALT activity.^9^,^10^ The utility of these markers at timepoints later than 48 hours is also highlighted in these studies, given their prognostic utility in late-presenting patients with established liver injury.

In summary, at first presentation to the hospital, miR-122, HMGB1, and necrosis K18 are more sensitive than ALT at identifying acetaminophen-induced acute liver injury. They have the potential to refine patient care in a number of ways. Future clinical trials of new therapeutic strategies could include one or more of these markers in the patient inclusion criteria with the objective of targeting new therapies or management pathways in lower or higher risk patients. If such trials were successful, more individualized treatment would be possible at the hospital “front door,” as is already the case with the management of acute coronary syndromes (being stratified by sensitive cardiac biomarkers such as troponin). Currently, patients who have taken a single acetaminophen overdose, who present late to the hospital, can only have liver injury confidently excluded if their liver function tests are normal around 24-36 hours after drug ingestion. The new biomarkers described in this article may allow earlier exclusion of injury, which would have an impact on hospital bed occupancy and avoid adverse acetylcysteine reactions by reducing unnecessary treatment. Patients with a timed blood acetaminophen concentration below the at-risk line on the treatment nomogram are usually not treated with acetylcysteine. Measurement of more sensitive markers, such as miR-122 and HMGB1 alongside ALT in these patients may increase physician confidence in discharging the patient from the hospital, although this requires a specific study in this group, as all the patients in the present study received treatment. Despite patients in the present study developing ALI, none reached the KCC for transplantation, received a liver transplant or died because^22^ 70% of our cohort was admitted to the hospital within the time frame required where prompt AC treatment is expected to be near 100% effective. It remains to be determined if the new biomarkers can predict these important clinical outcomes at first presentation to the hospital.

## Abbreviations

AC: acetylcysteine
ALI: acute liver injury
ALT: alanine aminotransferase
HMGB1: high mobility group box-1
GLDH: glutamate dehydrogenase
INR: International Normalized Ratio
K18: keratin-18
miRNA: microRNA
ROC: receiver operating characteristic
